# Health care utilization of mexican patients with medically unexplained physical symptoms

**Published:** 2016-09-30

**Authors:** Azucena Maribel Rodriguez González, José Manuel Ramírez Aranda, Homero de los Santos Reséndiz, María Yolanda Lara Duarte, Santiago Oscar Pazaran Zanella, Jafet Felipe Méndez López, Issa Gil Alfaro, Félix Gilberto Islas Ruz, Gloria Navarrete Floriano, Edith Guillen Salomón, Obdulia Texon Fernández, Silvia Cruz Duarte, Juan Carlos Romo Salazar, Claudia Elsa Pérez Ruiz, Sara de Jesús López Salas, Lizbeth Benítez Amaya, Javier Nahum Zapata Gallardo

**Affiliations:** 1 Red Mexicana de Investigadores en Medicina Familiar A. C. Monterrey, Nuevo Leon Mexico,; 2 Secretaria de Salud de Nuevo León Monterrey, Nuevo Leon, México; 3 Hospital Universitario "Dr. José Eleuterio González", Departamento de Medicina Familiar, Universidad Autonoma de Nuevo Leon, Facultad de Medicina, Monterrey, Nuevo Leon, México; 4 Colegio Mexicano de Medicina Familiar, Ciudad de Mexico, Mexico; 5 Facultad de Medicina de Tampico, Universidad Autónoma de Tamaulipas, Tamaulipas, Mexico; 5 Instituto Mexicano del Seguro Social, Ciudad de Mexico, Mexico

**Keywords:** Somatization disorder, somatization, healthcare institutions, healthcare services, primary health care, family medicine, ambulatory care, physician-patient relations

## Abstract

**Objective:**

To determine the prevalence of medically unexplained physical symptoms and the characteristics and use of health services in a group of patients with medically unexplained physical symptoms and a group of patients with other illnesses.

**Methods:**

This was a cross-sectional, retrospective and multicenter study. We included 1,043 patients over 18 years of age from 30 primary care units of a government health institution, in 11 states of Mexico, attended by 39 family physicians. The prevalence of medically unexplained physical symptoms was determined and both groups with or without symptoms were compared with regard to drug use, laboratory and other studies, leaves of absence, and referrals in the last six months. The group with medically unexplained physical symptoms was diagnosed using the Patient Health Questionnaire and the diagnostic criteria of Reid *et al*. Emergency or terminal illnesses were excluded. The chi square test was used with a statistical significance of p < 0.05.

**Results:**

Medically unexplained physical symptoms was diagnosed in 73 patients (7.0%). The majority were women (91.8%); their predominant symptom was from the gastrointestinal system in 56 (76.7%). This group had a greater use of clinical studies and referrals to other services (mean 1.1 vs. 0.5; *p* <0.0001 and 0.6 vs. 0.8; *p* < 0.01, respectively).

**Conclusions:**

The prevalence of medically unexplained physical symptoms was low, but with a greater impact on some health services. This could represent an overload in medical costs.

## Abstract (es)

**Objetivo:**

Determinar la prevalencia de Síntomas Físicos Medicamente No Explicables y las características y uso de los servicios de salud entre el grupo de pacientes con Síntomas Físicos Medicamente No Explicables, y el grupo con otras enfermedades.

**Métodos:**

Estudio transversal, retroprospectivo y multicéntrico. Se incluyeron a 1,043 pacientes mayores de 18 años, en 30 unidades de atención primaria de una institución gubernamental en salud, en 11 estados de la República Mexicana, atendidos por 39 médicos familiares. Se estimó la prevalencia de Síntomas Físicos Medicamente No Explicables y se compararon los dos grupos con y sin estos síntomas, en cuanto al uso de medicamentos, estudios de laboratorio, de gabinete, incapacidades y referencias en los últimos seis meses. El grupo de Síntomas Físicos Medicamente No Explicables fue diagnosticado por el Patient Health Questionnaire (son los síntomas físicos más comúnmente referidos por estos pacientes en el primer nivel de atención), además de criterios diagnósticos de Reid *et al*. Se excluyeron urgencias o con enfermedad terminal. Se utilizó prueba Chi cuadrada con *p* <0.05 para significancia estadística.

**Resultados:**

El 7.0% (73) se diagnosticó como Síntomas Físicos Medicamente No Explicables, la mayoría mujeres (91.8%); el síntoma predominante pertenece al sistema gastrointestinal con 76.7% (56). Este grupo demandó mayor uso de estudios de gabinete y referencias a otros servicios (media 1.1 vs. 0.5; *p* <0.0001 y 0.8 vs 0.6; *p* <0.01, respectivamente).

**Conclusiones:**

La prevalencia de Síntomas Físicos Medicamente No Explicables fue baja, pero con impacto significativo en el uso de algunos servicios de salud. Esto pudiera representar un mayor costo comparado con otro grupo de pacientes.

## Introduction

Patients with medically unexplained physical symptoms (MUS) are those in which no organic pathology is found that explains the origin of their symptoms 1. Its prevalence in primary care ranges between 1.1% and 15.3% 2-4; in secondary level care it is 52%, with a greater frequency in certain services 5. In Mexico, there are no data on its prevalence.

This variation in prevalence depends on differences in the population, on more or less stringent criteria for diagnosis, and the different names this disease has received, such as "somatization", "functional presentation", among others 6. 

Due to the difficulty in conclusively identifying patients with MUS, diagnostic criteria have been developed, such as that of Smith and Dwamena 7, the Diagnostic and Statistical Manual of Mental Disorders in Primary Care (DSM-IV-PC)1, Diagnostic and Statistical Manual of Mental Disorders, Fifth Edition (DSM-5) 8, the criteria by Steven Reid *et al*. 9, and the Patient Health Questionnaire (PHQ-15) 10.

The characteristics of patients with MUS may be related to the increase in the use of health services reported in the literature 2,11, since these patients have a reduced quality of life, and high disability rates 12. This impacts the cost of services provided, and causes a loss of productivity 13.

These patients are commonly seen by primary care physicians. With these physicians they generate a diagnostic and therapeutic challenge because their symptoms are not consistent with common clinical conditions, and for physicians with little experience, their diagnosis involves further laboratory and other tests. In addition, many patients have a strong emotional component to the disease, recurrent symptoms, and poor treatment adherence, problems that cause overutilization of health services.

Because of this, the aim of this study is to determine the prevalence of MUS in an outpatient setting and analyze the characteristics and use of health services between a group of patients with MUS and a group of patients with other ailments (not MUS). 

## Material and Methods

This was a cross-sectional, retrospective and multicenter study of a primary care outpatient population. The study population consisted of patients over 18 years of age cared for by 39 family physicians from 30 primary care public institution outpatient clinics located in 11 states of Mexico.

The data collection instrument of the study consists of a section of sociodemographics, a screening for MUS (PHQ-15), and characteristics of care. This vas applied during the period of September to December 2014. The physician randomly selected one patient per day. The aim of the study was explained and the patient then provided written informed consent. The patient answered the self-assessment sociodemographic data section and the screening section in the waiting room; after entering the the doctor´s office, the physician would fill out the diagnostic criteria for MUS. At the end of the working day, the doctor would complete the section on characteristics of care, using the patient´s electronic record. The completed instrument was sent to the researcher via email.

### Diagnosis of MUS

The diagnostic criteria for detecting somatic symptom were three or more unspecific symptoms according to the PHQ-15 (screening section), which are the first 15 questions of the PRIME-MD (Primary Care Evaluation of Mental Disorders), which correspond to the most commonly reported physical symptoms in primary care. This questionnaire has good consistency and moderate reliability (Cronbach's alpha 0.80 and a test-retest reliability of 0.60, respectively) 10, but due to its low sensitivity and specificity compared to clinical diagnosis 15, and for the aims of this research, we also considered the diagnostic criteria of Reid et al. (kappa index 0.76-0.88), to be diagnosed as MUS. These criteria are having evidence of investigation of nonspecific symptoms, negative test results, and having a psychosocial factor that suggests the presence of a symptom or diagnosis of a medically unexplained syndrome (fibromyalgia, irritable bowel syndrome, etc.) 9.

Patients who consulted for symptoms different from those listed on the PHQ-15 or with unspecific symptoms identified on the PHQ-15 or who did not meet the diagnostic criteria of Reid et al. were considered the non-MUS group. Patients who required emergency consultation and terminally ill patients were excluded.

### Sociodemographic data 

Were collected and the type of family of the patient was categorized according to composition and history of the use of healthcare services in the last 6 months, such as number of consultations, accumulated days of sick leave, drugs prescribed, consultations with specialists, laboratory and other tests (plain film or contrasted x-rays).and referral to secondary level care if further studies were needed.

The instrument was piloted with 20 patients to assess its consistency and to validate the estimation of sample size. 

The lack of consensus on the use of a single instrument for diagnosis of MUS is a bias in determining the prevalence, and consequently the comparison between the different sample sizes. However, as mentioned before, we preferred strict criteria for diagnosis. Despite the standardization of procedures, it is possible that there was some variability because it is a multicenter study.The sample was estimated to be 1,054 with a power of 0.90 to detect an effect size (d) of 0.20 (d= m1-m2/s) by using Student *t* test with a significance level of 0.05 when the alternative hypothesis is bilateral.

Percentages and frequencies were determined for categorical variables and measures of central tendency and dispersion for continuous variables. The Chi-square test was used with a *p* <0.05 for statistical significance in the case of categorical variables. After determining a normal distribution, Student *t* test or the Mann-Whitney test was used. The data was processed with SPSS v.20. Lost data were scarce and no special intervention was used for this data.

The study was approved by the Ethics and Research Committee of a university wih registration number R-2013-785-046.

## Results

This study included 1,050 patients (seven were eliminated because of incomplete data). In 7.0% (73) MUS was diagnosed. The sociodemographic characteristics of the patients are shown in Table 1 and the use of health services in Table 2. 

**Table 1.** Demographic data of patients (n = 1,043)

Characteristics	Without MUS* (n= 970)	With MUS* (n= 73)
Female sex**	697 (71.9)	67 (91.8)
Married**	597 (61.7)	44 (60.3)
Bachelor´s degree or greater**	444 (45.8)	50 (68.5)
Nuclear family**	599 (62.3)	61 (83.6)
Employed**	368 (38.2)	35 (48.6)
Urban area**‡	845 (87.4)	50 (68.5)
Age, years, mean (SD)	52.7 (15.1)	50.6 (15.8)

*n(%)

**p <0.05; SD= standard deviation.

‡Population with more than 2,500 inhabitants according to the Instituto Nacional de Estadística y Geografía (INEGI).

MUS: medically unexplained physical symptoms

**Table 2.** Use of health services in a family medicine practice in the last 6 months.

Variable	Without MUS n = 970	With MUS n = 73	p value*
Number of previous consultations in six months	2.9 (1.8)	1.8 (1.4)	0.000
Drugs prescribed	6.6 (6.3)	4.6 (5.2)	0.003
Days of sick leave accumulated	0.8 (3.9)	0.4 (1.8)	0.449
Laboratory studies requested	1.6 (1.8)	1.9 (1.5)	0.104
Clinical studies requested	0.5 (0.7)	1.1 (0.6)	0.000
Referrals to seondary care	0.6 (0.7)	0.8 (0.5)	0.003

MUS: medically unexplained physical symptoms

Data are shown as means ± standard deviation.

*p value= Mann Whitney U test

Forty percent (418) patients had three or more symptoms on the screening list (PHQ-15); the most frequent symptom was back pain in 236 (28.6%), followed by arm and leg pain in 136 (16.5%). In those with the diagnostic criteria of Reid *et al*., 7.0% (73), gastrointestinal system symptoms predominated in 76.7% (56), and in descending order, musculoskeletal 16.4% (12) and nervous system 6.9% (5); therefore, this were considered the MUS group.

The characteristics of symptoms in patients from the MUS group were recurrent in 79.5% (58). The patient had consulted before for this same symptom in 93.2% (68) of cases. Some other characteristics according to the judgment of the treating physician are shown in Table 3. 

**Table 3.** Characteristics of MUS according to treating physician´s judgement (n = 73).

Characteristics	With MUS*	
It was necessary to request laboratory studies and clinical studies during this consultation	Yes	71 (97.3)
	No	2 (2.7)
There is a logical correspondence between the symptoms and a defined clinical picture	Yes	40 (54.8)
	No	33 (45.2)
The patient has a psychological problem	Yes	11 (15.1)
	No	62 (84.9)

MUS: medically unexplained physical symptoms

*n(%)

With regard to comorbidity, the presence of chronic diseases, mainly chronic degenerative, was similar in both groups with 35.6% (345 patients without MUS and 26 with MUS). 

## Discussion

In this study, the prevalence of patients with MUS was below the range mentioned in other publications 4; this may be because strict diagnostic criteria were used to define MUS. The wide variability of prevalence could be associated to different diagnostic criteria and the lack of a unique concept 6. 

The number of references to secondary level care of patients with MUS was similar to that reported in several comparative studies 11,16. This could be related to uncertainty in their diagnosis and because their somatic complaints were not resolved by the primary care physician. 

Gastroenterology consultation showed a higher rate of referred patients, as mentioned in other studies17; this coincides with the most common symptoms found in patients who met the two diagnostic criteria used in this study 10,11. 

The same could be said about overuse of imaging studies in this group, which coincides with that published by Reid *et al*. 11, and Kinderman *et al*. 18, which may be related to insistence in finding the origin of symptoms for the patients or the search for differential diagnoses of the disease by the physician, aspects that can definitely influence the cost of healthcare, although in this research, a direct cost estimation of services used was not performed. There are publications that report elevated healthcare costs 16, which increase even more if the impact on labor and productivity are considered 12.

A contradictory fact is that there were more accrued days of sick leave in patients without MUS, although this was not statistically significant.

There was a similar finding with respect to laboratory tests requested. Those showed a higher trend in patients with MUS without statistical significance. In fact, in a study of patients with MUS, it was found that 21.6% of patients required studies 3. 

With regard to drug prescription, this was lower in patients with MUS. According to Salmon et al., patients without MUS usually request psychological support 19, or use effectively proven techniques such as cognitive behavioral therapy 20. Nevertheless, this could be because patients with MUS are referred to other levels of care to search for an explanation for their symptoms as mentioned before.

 Although in Norway no statistical significance was reported between the number of consultations for persistant MUS and the total number of consultations during the month 3, in this research, there was a lower number of consultations in MUS patients ( 1.8 vs 2.9) , a finding that could be related to the fact that the largest percentage of patients without MUS are individuals with chronic conditions who periodically attend to follow up medical appointments generating more consultations 21. 

The MUS population in this study was similar to that reported in the literature. Most were women, 91.8% (67), which coincides with published reports 2,5, and most were married 60.3% (44), as has also been reported 13,22. Mean age was 50.6 years (SD 15.8) while in other research, the mean is 48 years 3.

Most patients were employed, 48.6% (35), with this coinciding with other reports 3,5,22, and with a higher level of education, similar to that reported in the literature 12,5. This could be related, in this reseach, to the fact that the study was performed in workers who have health care coverage.

In this study, most patients came from a nuclear family; however, a study of patients with MUS constantly referred to secondary care reported a tendency to live alone compared to controls, although most of the people in the sample were married or lived in common law marriage 2. Again, this disparity or similarity in the findings of this research with the literature should be viewed as a lack of clarity in defining these patients 23, and of diagnostic protocols 24. 

The significance of the study is limited by the fact that there are no established diagnostic and screening criteria to identify patients with MUS. Finally, the use of health services and patient comorbidity was assessed in the clinical history from the last 6 months and therefore only represents a partial view of the impact on institutional outpatient care.

The strengths of this study reside on the fact that it was a multicentric study, conducted in various states of the Mexican republic by family physicians and that it represents the beginning of this line of research in Mexico.

 We suggest reaching a consensus to establish diagnostic and care protocols for patients with MUS. It is essential for the scientific community to reach an agreement about the best diagnostic tool for patients with MUS, in order to make treatment decisions and control unnecessary costs.

## Conclusion

Patients with MUS cause a greater impact on some health services in institutional outpatient clinics, this could represent an increase in medical costs. Identification and effective treatment of this group of patients is paramount.
